# A study protocol of a three-group randomized feasibility trial of an online yoga intervention for mothers after stillbirth (The Mindful Health Study)

**DOI:** 10.1186/s40814-017-0162-7

**Published:** 2017-07-06

**Authors:** Jennifer Huberty, Jeni Matthews, Jenn Leiferman, Joanne Cacciatore, Katherine J. Gold

**Affiliations:** 1 School of Nutrition and Health Promotion, Arizona State University, 500 N. 3rd St, Phoenix, AZ 85004 USA; 2 Colorado School of Public Health, University of Colorado Denver, 13001 E. 17th Place, B119, Bldg 500, Room E3341, Anschutz Medical Campus, Aurora, CO 80045 USA; 3 School of Social Work, Arizona State University, 411 N. Central, 8th Floor, Phoenix, AZ 85004 USA; 4 Department of Family Medicine, Department of Obstetrics & Gynecology, University of Michigan, 1018 Fuller Street, Ann Arbor, MI 48104-1213 USA

**Keywords:** Mental health, Perinatal loss, Women, Complementary, Mindfulness

## Abstract

**Background:**

In the USA, stillbirth (in utero fetal death ≥20 weeks gestation) is a major public health issue. Women who experience stillbirth, compared to women with live birth, have a nearly sevenfold increased risk of a positive screen for post-traumatic stress disorder (PTSD) and a fourfold increased risk of depressive symptoms. Because the majority of women who have experienced the death of their baby become pregnant within 12–18 months and the lack of intervention studies conducted within this population, novel approaches targeting physical and mental health, specific to the needs of this population, are critical. Evidence suggests that yoga is efficacious, safe, acceptable, and cost-effective for improving mental health in a variety of populations, including pregnant and postpartum women. To date, there are no known studies examining online-streaming yoga as a strategy to help mothers cope with PTSD symptoms after stillbirth.

**Methods:**

The present study is a two-phase randomized controlled trial. *Phase 1* will involve (1) an iterative design process to develop the online yoga prescription for phase 2 and (2) qualitative interviews to identify cultural barriers to recruitment in non-Caucasian women (i.e., predominately Hispanic and/or African American) who have experienced stillbirth (*N* = 5). *Phase 2* is a three-group randomized feasibility trial with assessments at baseline, and at 12 and 20 weeks post-intervention. Ninety women who have experienced a stillbirth within 6 weeks to 24 months will be randomized into one of the following three arms for 12 weeks: (1) intervention low dose (LD) = 60 min/week online-streaming yoga (*n* = 30), (2) intervention moderate dose (MD) = 150 min/week online-streaming yoga (*n* = 30), or (3) stretch and tone control (STC) group = 60 min/week of stretching/toning exercises (*n* = 30).

**Discussion:**

This study will explore the feasibility and acceptability of a 12-week, home-based, online-streamed yoga intervention, with varying doses among mothers after a stillbirth. If feasible, the findings from this study will inform a full-scale trial to determine the effectiveness of home-based online-streamed yoga to improve PTSD. Long-term, health care providers could use online yoga as a non-pharmaceutical, inexpensive resource for stillbirth aftercare.

**Trial registration:**

NCT02925481

## Background

In the USA, the death of a baby to stillbirth (in utero fetal death ≥20 weeks gestation) is a major public health issue. Each year more than 26,000 babies are stillborn, and in many cases, even after a postmortem evaluation, the cause is unknown [1]. Many mothers suppress their grief and feel stigmatized, socially isolated, and their baby’s lives devalued by society [2]. These experiences seem to worsen trauma reactions, such as despair, thoughts of suicide, anxiety, guilt, shame, and anger [3–5]. Symptoms can last up to 18 years [6–8] and are likely to re-occur in subsequent pregnancies [9, 10]. In an effort to destigmatize stillbirth, *The Lancet* has published two issues dedicated entirely to stillbirth in 2011 and 2016 [11, 12].

### Psychological health

Women who experience stillbirth, compared to women with live birth, have a nearly sevenfold increased risk of a positive screen for post-traumatic stress disorder (PTSD), a fourfold increased risk of depressive symptoms, twice the risk of a positive screen for generalized anxiety disorder [13, 14]. Symptoms of PTSD and its associated comorbidities (e.g., anxiety and depression) may contribute to diminished self-compassion (i.e., poor self care), emotional dysregulation, and compromised sleep quality, all of which have been associated with declining physiological well-being, weight retention/gain, increased chronic disease risk (e.g., heart disease, diabetes), premature mortality, poor quality of life, and cognitive delays in subsequent children [15–17]. Because the majority of women who have experienced the death of their baby become pregnant within 12–18 months [18], novel approaches targeting physical and mental health, specific to the needs of this population, are critical.

### Interventions

A recent Cochrane review [19] demonstrates the need for clinical trials to assess interventions to support women after the loss of a baby to stillbirth. A recent systematic review [20] evaluated intervention studies in bereaved mothers after stillbirth and identified only two [one small RCT and one feasibility study (outside of US)] from 1980 to 2015. One RCT evaluated a support group in women after a stillbirth compared to a no-treatment concurrent control group. The authors concluded that grief counseling may reduce grief symptomology after a stillbirth. This is particularly salient in mothers with poor social support [21]. The other explored the feasibility, acceptance, and cultural fit of a brief mindfulness-based intervention to reduce perinatal grief and other mental health indices (i.e., depression, coping) among Indian mothers [22]. There were significant improvements in anxiety, depression, coping, life satisfaction, social provisions, and mindfulness as a result of the intervention.

Given the lack of intervention studies conducted within this population, more research with appropriate control groups are needed to inform evidence-based care.

### Rationale for study

The most common treatments for PTSD are exposure therapy [23], eye movement desensitization and reprocessing (EMDR) [24], referral to support groups/interpersonal counseling [25, 26], and/or psychiatric medication or other medications to treat physical symptoms [27]. Although these strategies demonstrate some effectiveness in the general population with PTSD, they are unlikely to be fully effective for bereaved mothers for a variety of reasons. First, bereaved mothers’ engagement in treatment (e.g., initiation of therapy, support groups) is uncommon (<25%) [13]. Second, because bereaved mothers may be trying to conceive again, or have already conceived, they are often reluctant to take psychiatric medication, preferring non-pharmacologic alternatives [1]. In addition, medication cannot address the psychosocial aspects of grief and the accompanying emotional distress [28]. Third, though support groups for bereaved parents may provide a familiar and safe environment to connect with others who have had similar experiences [29, 30], these groups often have little to no emphasis on adaptive coping strategies for PTSD symptoms (e.g., hypervigilance, intrusion, avoidance) and the underlying emotional etiology [19, 31]. Fourth, bereaved mothers report unique barriers to participation in activities outside the home, such as encountering other babies in public, and many express strong interest in home-based treatments [32]. Finally, women may not have access to specialized treatment (e.g., exposure therapy, support groups) based on where they live, insurance coverage, or access to specialized clinicians, resulting in limited options for treatment [30, 33, 34]. It is crucial for science to develop novel approaches to inter-conception care amenable to the needs of these mothers.

Yoga may be an ideal treatment for addressing the unique PTSD symptoms of bereaved mothers after the death of a baby. Several studies have demonstrated that yoga is efficacious, safe, acceptable, and cost-effective for improving mental health in a variety of populations, including pregnant and postpartum women [35–41]. Yoga has also been used as a means to cope with PTSD symptoms after traumatic events (e.g., interpersonal violence, war, natural disasters) [42–45]. To date, there are no known studies examining yoga as a strategy to help bereaved mothers cope with PTSD symptoms after stillbirth.

This manuscript will describe a study protocol for a three-group randomized feasibility trial in US-based mothers and follows the reporting guidelines outlined by the SPIRIT checklist [46]. This study has the following objectives:

1. Examine the feasibility and acceptability of a 12-week, home-based, online-streamed yoga intervention, with varying doses (low = 60 min/week; moderate = 150 min/week) among bereaved mothers who have experienced the death of a baby to stillbirth. Feasibility is defined as acceptability, demand, and practicality (see the “Outcome measures” section for operational definitions).
2. Adapt an already established, evidence-based control group [47–50] and determine its acceptability for a future randomized controlled trial. Adaptation is defined as age- and population-specific. Acceptability is defined as adherence and satisfaction.
3. Ascertain the preliminary effects of the intervention on reducing PTSD symptoms below the clinical threshold.
4. Explore potential mechanisms (i.e., self-compassion, emotional regulation, sleep disturbance) by which yoga may reduce PTSD symptoms in bereaved versus non-bereaved mothers. We will also determine intervention effects on co-morbid conditions such as anxiety and depression.

## Methods/design

### Study design

**Fig. 1:**
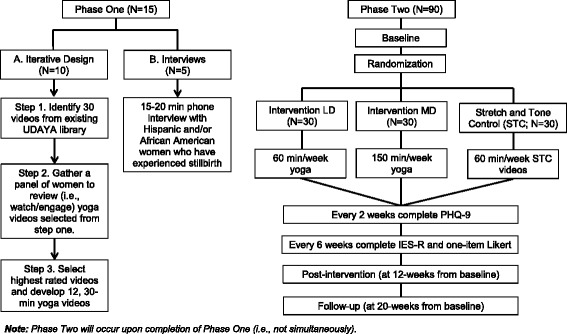
Study flow chart: the *left side* of the diagram illustrates the flow of the study for phase 1, and the *right side* illustrates the phase 2

The study will occur within two phases—one for the formative work to inform the intervention and another to implement the intervention (see Fig. 1 for a flow chart of study phases).

#### Phase 1

Part A. Iterative design: We will develop the yoga prescription using an iterative design process with the following steps:

1. Identify 30 videos from the existing UDAYA (an online-streaming commercial yoga website; https://udaya.com) library.
2. Gather a panel of women whose babies were stillborn (*n* = 5) and a panel of women who are regular yoga practitioners and have not experienced a baby’s death (*n* = 5) to review (i.e., watch or engage) yoga videos selected from step one above.
3. Develop (produced by Udaya.com) up to 12, 30-min yoga videos specifically for the grieving mothers.

Part B. Interviews to identify cultural barriers to recruitment: We will recruit five non-Caucasian (predominately Hispanic and/or African American) women who have experienced stillbirth to participate in a 15–20 min phone interview. These interviews will guide the recruitment process for phase 2, and additional recruitment materials will be developed to ensure adequate enrollment of non-Caucasian women in the study. The interview questions will consist of questions related to:

1. Perceptions/opinions about yoga related to physical and mental health.
2. Strategies to ensure adequate enrollment and retention of non-Caucasian women to participate in a yoga intervention.

#### Phase 2

Phase 2 is a three-group randomized feasibility trial with assessments at baseline and at 12 and 20 weeks from baseline. After completion of the consent process and the baseline assessment, 90 women will be randomized into one of the following three arms for 12 weeks: (1) intervention low dose (LD) = 60 min/week yoga (*n* = 30), (2) intervention moderate dose (MD) = 150 min/week yoga (*n* = 30), or (3) stretch and tone control (STC) group = 60 min/week of stretching/toning exercises (*n* = 30).

### Eligibility criteria

Inclusion and exclusion criteria for phase 1 (iterative design and interviews) are described in Table 1, and those for phase 2 are described in Table 2.

**Table 1:** Phase 1 eligibility criteria

Inclusion
Iterative design
• Able to read/understand/speak English
• Women who experienced stillbirth within past 6 weeks to 24 months OR
• Women who have not experienced stillbirth but have given at least one live birth who regularly practice yoga (participate in yoga >60 min/week)
Interviews
• Able to read/understand/speak English
• Racial/ethnic minority women
• Women who experienced stillbirth within past 6 weeks to 24 months
• Not regularly practicing yoga (participating in yoga <60 min/week)
• Underactive (≤120 min/week moderate-intensity physical activity)
Exclusion
Iterative design
• Women who have experienced stillbirth that regularly practice yoga (i.e., ≥60 min/week)
• Pregnant women
• Unstable psychiatric condition (psychosis; suicidal ideation with plan)
Interviews
• Caucasian women
• Unstable psychiatric condition (psychosis; suicidal ideation with plan)

**Table 2:** Phase 2 eligibility criteria

Inclusion
• Women who experienced stillbirth within past 6 weeks to 24 months
• Clinical levels posttraumatic stress symptoms (score of ≥33 on IES)
• ≥18 years of age
• Residing in the USA
• Able to read/understand/speak English
• Underactive (≤120 min/week moderate-intensity physical activity)
• Willing to be randomized
• Regular internet access via mobile phone, desktop/laptop computer, tablet, and etc.
• Answer “no” to all items on the PAR-Q (can participate safely)
Exclusion
• Unstable psychiatric condition (psychosis; suicidal ideation with plan)
• Pregnant at time of enrollment^a^
• Practicing yoga at least 60 min/week
• Unwilling to be randomized to a group
• Score of 20–27 of PHQ-9 (severe depression score)
• At risk for suicide based on follow-up phone assessment by Dr. Cacciatore after positive screen (PHQ-9 score of 1, 2, or 3)

^a^Pregnant women will not be eligible for initial recruitment into the study. Women who become pregnant during the study will complete a PARmed-X for Pregnancy with their physician to identify any contraindications to yoga and will be able to continue participation

### Interventions

#### Phase 1

##### Iterative design

Women (*N* = 10) will be asked to come to the Healthy Lifestyles Research Lab at a *University in the Southwestern United States* for nine sessions (i.e., 20–60 min)*.* Thirty online yoga videos from Udaya’s library, preselected by a yoga therapist and the principal investigator, will be randomly assigned (using a random number table) to participants. Each participant will be randomly assigned nine different online yoga videos so that each video is reviewed at least three times. Selection criteria for the 30 online yoga videos include the following: (1) have been used in our previous beta-testing [51] *and were* positively reviewed by mothers bereaved by stillbirth, (2) videos that the research team believes will cultivate emotional regulation and self-compassion (evidence-based proposed mediators from a general population with PTSD), (3) classes that are slower moving (i.e., beginner-based) with detailed instruction and alignment cues (i.e., safe and proper form), and (4) classes that are safe for women up to 20 weeks gestation in case a woman becomes pregnant during the intervention. Each woman will be asked to watch/engage in the yoga video by herself in an observation room. Participants will not be able to review more than one video per day to limit influence from another video. Before and after engaging in the yoga videos, women will be asked two investigator-developed questions related to the potential mechanisms by which yoga may reduce PTSD symptoms, including self-compassion [How much self-compassion do you feel (i.e., self-kindness, understanding toward yourself and your grief, common humanity, mindfulness) right now?] and emotional regulation [How much do you feel that you are able to be with your emotions (i.e., consciously stay with your emotions or mood) right now?]. Questions will be answered on a five-point Likert scale. Mean scores will be taken for each video and entered into an Excel document. Those videos that elicit the *highest* self-compassion and/or emotional regulation will *be used for phase 2*.

### Interviews

One research staff member trained by an expert in qualitative research will conduct the interviews using a semi-structured interview guide. All interviews (*N* = 5) will be recorded and transcribed verbatim. The insight provided from the interviews will be used to modify the recruitment materials for phase 2 as needed.

#### Phase 2

The online videos prescribed in all three conditions will be suitable for women up to 20 weeks gestation (the maximum possible based on our study eligibility) in the case that a participant becomes pregnant during the study. Additionally, a nationally certified instructor (i.e., yoga or fitness) will teach the online videos.

##### Low and moderate dose intervention groups

The low and moderate dose intervention groups will be asked to follow a 12-week online yoga prescription (for 60 and 150 min/week, respectively). We selected the low dose of 60 min/week because this is the minimal amount needed to experience mental and/or physical health benefits [40, 52–55]. We chose the moderate dose of 150 min/week because this reflects the physical activity guidelines [56].

The style of the online yoga videos will be Hatha because (1) Hatha combines a physical practice with a meditative component to cultivate mindfulness (i.e., moment to moment, non-judgmental awareness, cultivated by paying attention in a specific way) [57] and self-awareness and (2) includes instruction from a variety of lineages (e.g., Iyengar, Asthanga, Vinyasa). The online prescription assures the poses used are identical for both intervention groups. Participants will be asked to complete the yoga videos in a specific order, which will include specialized preparatory instructions for each video to ensure comfort and safety [56].

##### Stretch and tone control group

The STC group will be asked to follow a 12-week online stretching/toning exercise prescription for 60 min/week to match minimal dose of intervention group (i.e., LD). We will develop 12, 30-min videos for the STC group prescription (produced and filmed by UDAYA). The STC videos will be adapted from an already existing, well-established, evidence-based control condition (originally designed for older adults in other funded trials) to be specific for women who are underactive (<150 min of moderate activity per week) [47–49, 58, 59]. The instructor will begin and end each class with a 3-min warm-up/cooldown. The instructor will ask participants to perform each isolated stretch or toning exercise for 1–2 sets (20–45 s or between 10 and 15 repetitions). Participants will be asked to perform slow, controlled, and complete movements. Progression will include demonstration by the instructor of modifications with a slightly less difficult and a more challenging version for the specific exercise. For example, a participant may be asked to perform a plank on their knees and a modification may be to straighten their legs, making the exercise more difficult. Variability will be demonstrated by changing the stretching and toning exercises every 3 weeks. For example, in the first 3 weeks, participants will be introduced to the new activities, and in the remaining weeks 4 to 12, they are encouraged to increase the difficulty by adding more repetition or performing the more challenging modification. Participants will be asked to complete the STC videos in a specific order, which will include a warm-up, four upper body, four lower body, three trunk/core, and one to two balance exercises, with appropriate progression over 12 weeks. The STC videos will have discussion related to form and safety by the instructor (i.e., no guidance for movement with breath or mindfulness). Additionally, videos will have students in the background following instructor cues and demonstrating variations of exercises. There will be no final relaxation pose in the STC videos (i.e., the rest period after completion of a yoga class).

##### Procedures

UDAYA will provide the research team with 90 membership accounts pre-paid for the length of the study that will have access only to the videos specific to the participants group assignment (i.e., participants will not have access to the full UDAYA video library). The research staff will assign UDAYA membership accounts to all participants (i.e., intervention and control) upon enrollment into the study and will provide them with a username and password to access UDAYA prior to their participation in the study. In addition, participants will be asked to wear an accelerometer (i.e., GENEActiv) to measure physical activity and sleep quality for 1 week at baseline, post-intervention (12 weeks from baseline), and follow-up (20 weeks from baseline) and complete assessments at these time points.

Participation in the videos (intervention and control groups) will be automatically tracked (class taken, time of day, length of use, and etc.) via web analytics software throughout the study in each group. Research personnel will send weekly reminders (email or text message) to participants for minimal dose compliance. Additionally, the research team will send each participant a Qualtrics link to report (1) completion of sessions, (2) rating of perceived exertion, (3) assessment of mindfulness, (4) other physical activity participation, (5) pregnancy status, mental health, use of psychiatric medication, and adverse events. Participants will be asked weekly about participation in support groups or individual/group-based counseling sessions. Participants will be discouraged from attending face-to-face yoga/stretching/toning classes during the study.

In the case that a woman becomes pregnant during the study, she will be asked to complete the Physical Activity Readiness Medical Examination for Pregnancy (PARmed-X for Pregnancy), a health screening tool completed with the health care provider to ensure it is safe for women to participate in prenatal exercise. We will also send women a pregnancy safety handout (specific to yoga or general physical activity depending on group assignment) for their review.

The study will be stopped prior to its completion based upon (1) clinical deterioration of PTSD symptomology (increased scores of >20 points from baseline on the Impact of Event Scale-Revised (IES-R) in >50% of the enrolled participants), (2) difficulty in study recruitment (i.e., at halfway through the timeline of recruitment, study recruitment falls below 30% of the total recruitment goal), and (3) if any new safety information or research becomes available during the trial that necessitates stopping.

### Outcome measures

#### Phase 2

Table 3 outlines the outcome measures and associated time points.

**Table 3:** Summary of data collection

	Baseline	12 weeks	20 weeks
Demographics	X		
Feasibility (acceptability, demand, practicality)			
Interviews		X	X
Acceptability			
Satisfaction survey		X	
Demand			
Udaya.com tracking system	X	X	
Daily log	Daily		
Practicality			
Udaya.com tracking system	X	X	
Satisfaction survey		X	
PTSD symptoms			
Impact of Event Scale-Revised	X	X	X
Comorbid conditions			
State-Trait Anxiety Inventory (anxiety)	X	X	X
PHQ-9 Patient Health Questionnaire (depression)	X	X	X
Self-compassion			
Self-Compassion Scale	X	X	X
Emotional regulation			
Emotional Regulation Questionnaire	X	X	X
Grief			
Perinatal Grief Scale	X	X	X
Sleep quality			
GENEActiv	X	X	X
Pittsburgh Sleep Quality Index	X	X	X
Physical activity			
GENEActiv	X	X	X
Past-week Modifiable Activity Questionnaire	Weekly		
Daily log			
Yoga sessions	Daily		
Borg Rating of Perceived Exertion Scale	Daily		
Meditative Movement Inventory	Daily		
Self-rated health			
Short-Form 12 Health Survey	X	X	

##### Feasibility (acceptability, demand, and practicality)

We will utilize Bowen and colleagues’ [60] standards for feasibility studies. *Acceptability* measures include satisfaction with the online-streamed yoga intervention, perceived appropriateness of the intervention for this population, and intent to continue using intervention materials and will be assessed via satisfaction survey at post-intervention. *Demand*, including expressed interest in participating in study and actual use of intervention, will be measured via Udaya.com online tracking (i.e., # times logged in, time spent in class, completion of class, date/time of class access) and daily logs (i.e., yoga sessions, Borg Rating of Perceived Exertion Scale, and Meditative Movement Inventory). *Practicality* measures, including the ability of participants to carry out intervention activities (i.e., yoga), will be assessed via the Udaya.com tracking system, weekly logs, and satisfaction survey at the end of the intervention. *Qualitative interviews* following best practices for conducting interviews, including developing a priori questions and written moderator protocol and recording and transcribing data [61, 62], will be used to supplement information gained from the satisfaction surveys and to further understand the use of non-pharmacological interventions such as yoga in this population. Qualitative interviews will be administered in the intervention groups at post-intervention (12 weeks) and at follow-up (20 weeks).

For the intervention group(s), our benchmarks will be as follows: (1) feasibility (i.e., satisfaction, useful, appropriate, acceptable ≥70%, (2) demand (i.e., recommend to others, continue to use skills, program easy to complete ≥70%, and (3) attendance (i.e., at least 70% of participants in the moderate dose group will complete 150 min of yoga/week for at least nine of the 12 weeks (75%), at least 70% of participants in the low dose group will complete 60 min of yoga/week) for at least nine of the 12 weeks (75%). For the control group, our benchmarks will be as follows: (1) feasibility (i.e., satisfaction, appropriate, acceptable ≥70%) and (2) attendance (i.e., at least 70% of the control group will complete 60 min of stretching, toning, and limbering/week for at least nine of the 12 weeks (75%).

##### PTSD symptoms

The IES-R has three subscales (i.e., avoidance, intrusion, hyperarousal), and the sum of the three subscale scores produce the total IES-R score. All subscale scores have demonstrated high internal consistency and have been validated in postpartum populations [63]. A total IES-R score is clinically significant, and ≥33 signifies the likely presence of PTSD [64].

##### Anxiety

The State-Trait Anxiety Inventory (STAI) yields scores indicating levels of trait (Form Y-1) and state anxiety (Form Y-2). The STAI has demonstrated reliability and validity in pregnant and postpartum populations [65, 66]. Higher scores indicate greater anxiety.

##### Depression

The Patient Health Questionnaire-9 (PHQ-9) [67] is a valid and reliable (*α* = 0.86–0.89) measure used to screen, diagnose, monitor, and measure the severity of depression and has been validated in pregnant and postpartum populations [68–70]. Scores range from 0–27, and cut-off scores of 5, 10, 15, and 20 represent mild, moderate, moderately severe, and severely depressive symptoms, respectively.

##### Self-compassion

The Self-Compassion Scale (SCS) is a 26-item questionnaire with good construct validity and reliability (*α* = 0.92) [71]. Higher scores indicate higher levels of self-compassion.

##### Emotional regulation

The Emotional Regulation Questionnaire (ERQ) is a valid 10-item scale used to measure an individual’s tendency to regulate their emotions by two strategies (i.e., cognitive reappraisal and expressive suppression) [72]. The higher the scores, the greater the use of the emotional regulation strategy.

##### Mindfulness

The Mindful Attention Awareness Scale (MAAS) is a valid and reliable measure with good internal consistency (*α* = 0.80–0.87) [73]. This scale includes 15 items and measures the extent to which individuals are able to maintain awareness of present-moment experience. Higher scores reflect higher levels of mindfulness.

##### Grief

The Perinatal Grief Scale (PGS) is a valid 33-item instrument used to quantify the grief of perinatal loss [74]. The PGS consists of three subscales (i.e., active grief, difficulty coping, and despair). Higher scores indicate more intense grief.

##### Self-rated health

The Short-Form Health Survey (SF-12) was developed for the Medical Outcomes Study (MOS) and is modified from the original 36-item survey [75]. The SF-12 is a valid scale that includes 12 items that measure eight domains of health-related quality of life (i.e., physical functioning, role physical, bodily pain, general health, vitality, social functioning, role emotional, and mental health). The items are scored using standard scoring algorithms explained elsewhere [76]. Scores range from 0 (lowest level of health) to 100 (highest level of health).

##### Subjectively measured sleep quality

The Pittsburgh Sleep Quality Index (PSQI) is a 19-item questionnaire [77]. The PSQI has demonstrated reliability and validity in pregnant populations [78]. Higher global PSQI scores indicate more sleep disturbances.

Subjectively measured physical activity: The past-week Modifiable Activity Questionnaire (PWMAQ) is a valid and reliable self-report measure (*α* = 0.82) [79]. Respondents report the number of minutes spent in 38 different activities, and each activity is summed. Using the Compendium of Physical Activities, a MET value of >3.0 will be applied to each reported activity (at least a moderate-intensity). Participants will be categorized as meeting physical activity guidelines if they reported at least 150 min of moderate-intensity physical activity during the past week.

##### Objectively measured sleep quality and physical activity

The GENEActiv (ActivInsights, Kimbolton, UK) accelerometer will be used to provide an objective, non-invasive 24-h Assessment of Sleep-Wake and Physical Activity patterns. Wrist actigraphy has been extensively validated as a measure of sleep-wake activity as compared to the gold standard sleep measure, polysomnography, in samples from infants to older adults [80–82]. The GENEActiv also has been validated for sedentary behavior and postural allocation (i.e., sitting vs. standing), and moderate to vigorous physical activity [83, 84]. Bed times and wake times (to define the sleep interval) will be derived from brief daily diaries which can be completed in less than 1 min.

### Sample size

We aim to enroll 90 participants. Assuming an attrition rate of 20%, we will have a final sample of 72 women with data through a 20-week follow-up. This sample size is appropriate for early-phase developmental trials [60]. While we do not intend to fully power our exploratory aim 3, we do anticipate sufficient power to detect a large effect (to detect a large effect of Cohen’s *d* = .80) between a treatment arm and control group on primary (PTSD symptoms) and secondary (anxiety, depression, self-compassion, emotional regulation, sleep disturbance) outcomes with *n* = 72 (*p* < 0.05, power = 0.80). The power of the study is such that large effects will be detectable and the directionality of effects will be sufficiently stable to assess the preliminary merit of the intervention and provide information needed for future larger studies. Effect sizes will be calculated along with tests of significance. Effects of intervention on physical activity, mindfulness, and perceived exertion will be explored using mixed models or multilevel models to examine multiple waves of data from weekly logs and GENEActiv. Effects of intervention should emerge as significant regression coefficients for the group membership predictor in accounting for within-person intercepts and slopes. Planned contrasts will compare three groups (LD, MD, and control) for differing trajectories over time. If both yoga groups show greater improvements on outcomes than the control group and there are significant differences between LD and MD, this would indicate that one group may lead to improved outcomes. If both yoga groups improved more than the control group, and there are *no* significant differences between LD and MD, we will use feasibility data to determine which group is more feasible.

### Recruitment

We will nationally recruit and screen participants via a web-based survey (i.e., Qualtrics). We will work with non-profit partners who provide support for bereaved parents (e.g., MISS Foundation, Star Legacy Foundation) to actively recruit participants. We will also heavily utilize web-based strategies including social media (i.e., Facebook, Twitter), social networking sites, online stillbirth support groups (e.g., SOBBS, Compassionate Friends), and email LISTSERV. We will make a concerted effort to recruit minority women utilizing the information learned from the interviews with minority women in phase 1. Note: recruitment for the iterative design process in phase 1 will involve contacting *local* organizations following the same strategies above.

### Study enrollment

Interested participants will complete an eligibility screener (approximately 5–10 min) on Qualtrics. The screener will only allow one entry per IP address to prevent “ballot box stuffing.” Additionally, there will be a final question on the screener asking the participant to confirm all responses. Upon submitting the screener, participants will no longer have access to the screener. Research team members will follow an Institutional Review Board (IRB)-approved script to respond to interested participants who phone or email and refer them to the eligibility screener (participants provide consent electronically to complete the eligibility screener). All ineligible participants will be notified by phone and/or email and offered a discounted Udaya.com membership ($9/mo., compared to $12/mo.)

#### Phase 1

Iterative design: Eligible participants (see Table 1 for eligibility criteria) will receive an email requesting times to schedule their first visit to the lab on the university campus. During the participant’s first visit, they will complete an informed consent document.

Interviews: Eligible participants will receive an email requesting times to schedule their 15–20-min phone interview. Following, participants will be sent a Qualtrics link asking them to electronically sign an informed consent document before their scheduled interview.

#### Phase 2

Eligible participants (see Table 2 for eligibility criteria) will be asked to complete a phone intake appointment, an electronic informed consent, a HIPAA authorization form, and baseline assessments of self-report measures (see Table 3). Participants will then be randomized to an intervention (LD or MD) or control group. The research team will mail intervention group participants: (1) a packet with study info and directions, (2) a document including strategies to overcome barriers to online yoga participation, (3) an accelerometer (GENEActiv) with instructions on how to wear, and (4) a yoga mat, two blocks, and a strap. The control group will receive (1) a packet with study info and directions, (2) a document including strategies to overcome barriers to online physical activity participation, (2) an accelerometer (GENEActiv) with instructions on how to wear and a pre-paid return mailer, and (3) a stretching mat and band. All participants will be asked to begin wearing the GENEActiv immediately upon receiving it for one full week and then mail monitor back in a pre-paid envelope supplied in their packet. The GENEActiv will be mailed to participants again at post-intervention (12 weeks from baseline) and follow-up (20 weeks from baseline) using the same process. When participants have worn the GENEActiv for 1 week, they will be emailed their UDAYA username and password to begin accessing their respective videos.

### Confirming participants’ identity/eligibility

During the intake phone call, we will confirm the participant’s identity and eligibility with the following:

1. Confirmation of answers from the eligibility screener (i.e., date of the stillbirth delivery, cause if known, location of death).
2. A small sample of women (~30%; *N* = 27) will be asked to verify information about their baby’s death to confirm their eligibility based on stillbirth history. All women will be asked to sign a HIPAA authorization form at the same time in which they sign their informed consent (in the case where they are randomly selected for research staff to obtain medical records related to the death of their baby). As women enter the study and are assigned an ID, those with an ID that matches one contained in a random number table generated prior to recruitment into the study will be selected.
3. The research team will request the records (e.g., date, gestational age, birthweight, and Apgar scores). If there is a significant number of women who are ineligible (>30%), we will increase the number that we confirm eligibility to ~40% using medical records.

### Allocation (phase 2 only)

**Fig. 2:**
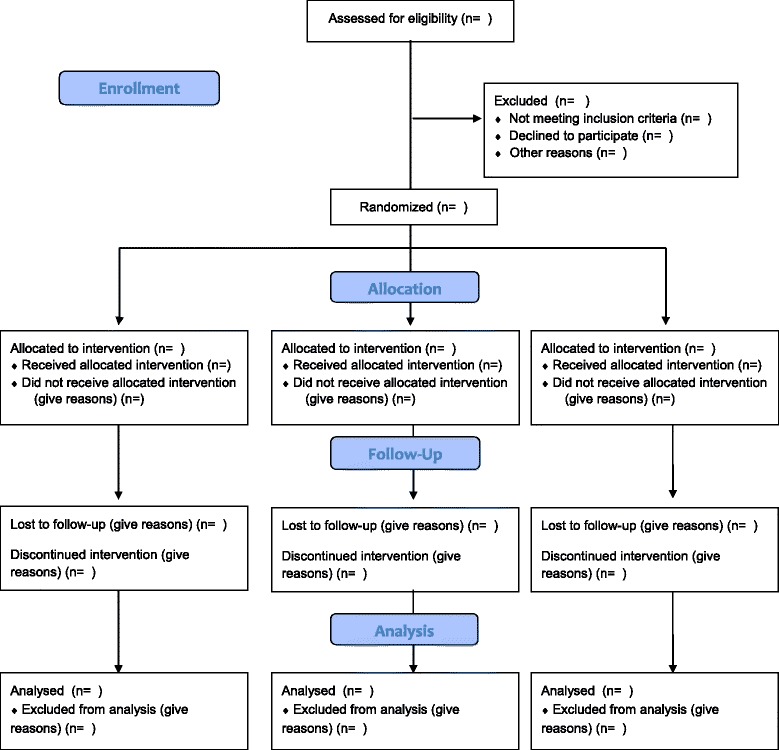
Phase 2 flow chart: This diagram illustrates a study flow chart adapted from the CONSORT and includes a blank template of what will be reported

Block randomization will be used to assign participants to treatment groups. Specifically, the biostatistician will construct 15 blocks of six participants using SAS PROC PLAN with a random number seed known only by the biostatistician. This will be done at the beginning of the study (front-loaded) and provided to a research staff member not associated with this study (i.e., trusted broker). The trusted broker will utilize the treatment allocations to sequentially assign enrollees to randomized treatment groups (the biostatistician will be shielded from enrollment date and enrollment order, and participant IDs will not reflect enrollment order). The trusted broker will hold the codes that match participants to treatment groups. Research staff members will call the trusted broker to determine assignment to group. In case of adverse events (AE), the research staff member will call the trusted broker to provide for unblinding (AE only). After randomization, participants will be emailed a study information packet (i.e., instructions, UDAYA username/password) according to the intervention assigned. The initiation of the study will begin after allocation to study arm (see Fig. 2 for phase 2 study flow chart).

### Blinding (phase 2 only)

The principal investigator and biostatistician will remain blinded until the completion of data collection (i.e., data is locked). Following the conclusion of data collection (i.e., data is locked), the biostatistician will be unblinded as data analysis will require knowledge of treatment assignment. The study coordinator and graduate student worker that deliver the intervention(s) and prepare reports to the Independent Monitoring Committee (IMC) will be unblinded. The study coordinator will be responsible for the intervention groups, and the graduate student worker will be responsible for the control group. A research staff not directly involved in the study will serve as the “trusted broker” to handle the sequential assignment of participants to randomized treatment groups and will also be unblinded.

### Incentives

Phase 1 and 2 incentives are listed in Table 4.

**Table 4:** Incentive schedule

Phase	Time point	Incentive
Phase 1—iterative design	Completion of review of 9 yoga videos	•Free 1 month membership to UDAYA •$15/session (up to $135 gift card)^b^
Phase 1—interviews	Completion of 15-min interview	$15 gift card^b^
Phase 2	Enrollment	•Intervention groups: yoga mat, 2 blocks, and strap •Control group: stretching mat and band •12-week membership to UDAYA^a^
	Baseline	Survey: $10
	Post-intervention	Survey: $10 Interview: $15
	Follow-up	Survey: $25 Interview: $10

^a^All groups receive incentive

^b^Participant has choice of gift card from Amazon, Bath & Body Works, Starbucks, or Target

### Data management

The research participant will self-report data electronically via Qualtrics. Weekly, the research team will be responsible for ensuring the completeness and timeliness of the data reported by the participants. Additionally, the research team will skim the data after each collection time point to ensure it is complete and will follow-up with any participants in which data collection is not complete.

Data quality will be monitored throughout the study. Measurement values will be compared to their known ranges. Out of range values will be investigated and either corrected if possible or treated as missing data. Statistical analyses will adjust for baseline demographic characteristics when statistically appropriate. Multiple imputations will be used to conduct statistical analysis with missing data. In the event that the missing at random assumption is violated, we will model the missing data mechanism and conduct sensitivity analysis on model parameters.

We have multiple strategies in place to identify yoga participation both during video sessions and outside of video sessions. First, we will be collecting 24-h, time-stamped GENEActiv accelerometer data at baseline, post-intervention (12 weeks), and follow-up (20 weeks) and will have a full record of their activity level during these time points. We will also be monitoring UDAYA website log-in information and will be able to localize activity monitor data while the participant is logged into the website and using the yoga videos. This will allow us to verify whether the participant is being active during the times they are using the website. Second, we will collect self-reported minutes of yoga using a log. This self-report log will also give us a time stamp of yoga activity that we will be able to cross-refer to the accelerometer data. Finally, we will be able to monitor all other physical activities that are performed outside of yoga, examining whether the intervention may have unintended effects of activity within other types of physical activity. Collectively, we will have a full record of yoga and physical activity (outside of yoga) participation that we will be able to use to understand the “dosage” that is being achieved for this trial.

### Statistical analysis

Inferential analyses: To examine quantitative feasibility measures, we will use Fisher’s exact tests when expected cell counts <5 and *t* tests to compare feasibility scores between the low and moderate dose groups. Significant differences between the two groups will indicate which yoga group is more feasible. We will also use logistic and Gaussian regression analyses to further compare feasibility scores between groups while adjusting for baseline participant characteristics.

We will use Fisher’s exact tests and ANOVAs to compare quantitative acceptability scores across control and yoga groups. Logistic and Gaussian regression analyses will be used to compare scores while adjusting for baseline participant characteristics. We expect participants in the control group to report similar levels of acceptability compared to those in the yoga groups, but participants in the yoga groups are expected to report greater improvements on outcomes than control participants.

Descriptive analyses: SPSS and *R* will be used to conduct descriptive statistical analyses, including graphical analysis (e.g., scatter plots, boxplots and histograms) and data summarization (e.g., means, medians, ranges, cross-tabulations) to identify data entry errors, outliers, and non-normal distributions. Standard errors and confidence intervals will be calculated using either parametric or non-parametric methodology, including the use of the bootstrap to estimate standard errors when parametric assumptions fail.

Data from the qualitative interviews will be transcribed and independently coded by the research team using NVivo. After coding is complete, researchers will meet to reach agreement on major themes of the interviews. Once the major themes have been determined, findings will be verified through member checking to determine accuracy of findings. Principal Investigator (PI) Huberty and Co-I Cacciatore have extensive training and experience in qualitative methods.

Psychometric analyses: We will evaluate scale reliabilities using Cronbach’s alpha coefficients based on baseline (T1) data. Scale inter-correlations will be estimated along with complete descriptive statistics on scale items.

Randomization check: We will also conduct an ANOVA to compare the experimental and control groups on outcome and demographic variables at baseline.

#### Missing data

Substantial missing data are anticipated in this study. Participants may refuse to participate or withdraw from the study at any time. In addition, daily and weekly logs may not be complete despite efforts by research staff to encourage completion. Missing data due to non-response on certain items within a scale will initially be handled by creating an average scale score for all participants who responded to at least 75% of items on that scale. We will evaluate whether missingness due to attrition is related to group membership and examine relations between missingness and any missing individual’s pretest scores on outcome variables. If missingness is found to satisfy the missing at random assumption, we will handle the missing data using multiple imputation. Multiple imputation will also be used to handle item-level missingness for final analyses of scales. In the event that the missing at random assumption is violated, we will model the missing data mechanism and conduct sensitivity analysis on model parameters. All analyses will retain all participants who were assigned to the intervention or control groups (i.e., intent to treat).

### Data monitoring

Due to the nature of this study, an IMC will be established and comprised of four committee members. Due to their combined expertise related to stillbirth care, biostatistics, NIH-funded studies, and yoga safety, they are qualified to review the patient safety data generated by this study.

#### Adverse events

An AE is any untoward medical occurrence in a subject during participation in the clinical study or with use of the experimental agent being studied. An adverse finding can include a sign, symptom, abnormal assessment (laboratory test value, vital signs, electrocardiogram finding, etc.) or any combination of these regardless of relationship to participation in the study. Adverse events that are non-serious will be provided to the IMC with summaries of the numbers and rates of AEs by group.

Expedited review will occur for all events meeting the FDA definition of serious adverse events (SAEs), i.e., any fatal event, immediately life-threatening event, and permanently or substantially disabling event. This also includes any event that a study investigator or the IMC judges to impose a significant hazard, contraindication, side effect, or precaution. For purposes of this study, all SAEs will be reported to the IMC regardless of any judgment of their relatedness to the study treatment.

The PI will record all reportable events with start dates occurring any time after informed consent is obtained until 7 (for non-serious AEs) or 30 days (for SAEs) after the last day of study participation. Each week, participants will report in their weekly logs the occurrence of AE/SAEs since the last week. If the participants are not completing their logs, they will be prompted via email by the research team to complete their logs. If there are two consecutive failed email attempts, the participants will be called. Events will be followed for outcome information until resolution or stabilization.

The research team will determine if the event is an AE or an SAE. If an SAE, the research team will complete a form to be provided to the IMC to meet and investigate the SAE until determined resolved or stable and its relation to the study (related or not related). Once recruitment begins, IMC will meet twice a year and will meet ad hoc when AE or an SAE occur. AE reports and annual summaries will not include individual identifiable private information about the human subjects. Each report will only include the identification code previously assigned.

At 6 weeks (midway through the intervention), participants will be asked to complete the IES-R and answer a 1-item Likert-based question related to whether they think the intervention is contributing to their symptoms for the IMC to review. Every 2 weeks of the intervention, participants will be asked to fill out the PHQ-9 via Qualtrics within 48 h. A research team member will monitor PHQ-9 completion daily (including weekend/holidays). Women with scores suggestive of clinical depression (>15) will be notified and advised to follow up with their primary care physician. If suicidal ideation is noted with a screen score of two or three on question 9 of the PHQ-9, the participant will be contacted within 24 h by a crisis-trained, clinical staff for further assessment of risk. Clinical providers trained by the MISS Foundation are well equipped to handle psychological crises, have a system currently used for their existing 3000 grieving families they help annually, and have staff available on weekends/holidays. If imminent suicidal risk is present, the staff will follow MISS Foundation emergency procedures to ensure participant safety, and the participant will be discontinued from the study. If there are no concerns for active suicidal risk after the phone assessment, the participant will be able to continue in the study if she desires. All participants will have the MISS Foundation provider’s phone number, and the research personnel can either email, call, text, or page the lead clinical provider to respond to psychiatric issues. Depending on the need of the participant, one of them will be able to talk to a clinician within 2 to 3 h.

If at any time during the study a participant should call a member of the research team emotionally distressed, not making clear sense, sounding intoxicated or impaired, or expressing thoughts of suicide or homicide, the research personnel will use a second phone line to contact the counselor, without losing verbal contact with the woman. At that point, the clinician will guide the interview to ascertain the acuity and/or potential for harm and will first determine if the caller is alone or has access to another adult capable of transportation. If determined that the woman is in need of emergent care, options for such care will be discussed with the caller and may include referral to the closest emergency room (ER), notification of emergency services for transportation to the nearest ER, and emergent counseling appointment coordinated by the MISS Foundation or their preferred provider. If the subject declines such a referral and the clinician remains concerned about the participant’s safety, emergency services in her area (determined during the consent process) will be notified of the clinical concern for safety.

#### Reporting

The PI will report any incidents, i.e., adverse events (AEs) or serious adverse events (SAEs), that meet the Office for Human Research Protections (OHRP) criteria for unanticipated problems. The PI will complete a form with the following information to the IRB: (1) appropriate identifying information for the research protocol (i.e., title, investigator’s name, IRB project number); (2) a detailed description of the adverse event, incident, experience, or outcome; (3) an explanation of the basis for determining that the adverse event, incident, experience, or outcome represents an unanticipated problem; and (4) a description of any changes to the protocol or other corrective actions that have been taken or are proposed in response to the unanticipated problem. Unanticipated problems that are serious adverse events will be reported to the IRB, IMC, and National Center for Complementary and Integrative Health (NCCIH) within 7 days, and any other unanticipated problem will be reported within 14 days of the investigator becoming aware of the problem/event. All unanticipated problems should be reported to the appropriate institutional officials (as required by an institution’s written reporting procedures), the supporting agency head (or designee), and the OHRP within 1 month of IRB’s receipt of the report of the problem from the investigator.

Study progress and safety will be reviewed monthly (and more frequently if needed). Progress reports, including patient recruitment, retention/attrition, AEs, and SAEs, will be provided to the IMC semi-annually. An annual report will be compiled and will include a list and summary of AEs and SAEs and will address (1) whether AE/SAE rates are consistent with pre-study assumptions, (2) the reason for dropouts from the study, (3) whether all participants met entry criteria, (4) whether continuation of the study is justified on the basis that additional data are needed to accomplish the stated aims of the study, and (5) the conditions whereby the study might be terminated prematurely. The annual report will be sent to the IMC and will be forwarded to the IRB and NCCIH. The IRB and other applicable recipients will review progress of this study on an annual basis. The study team will generate study reports for the IMC and will provide information on the following study parameters: enrollment status, subject status, demographics, stopping or halting rules, unanticipated problems, adverse events, protocol deviations, quality management, and outcome data (if applicable at time of report). Study report tables will be generated only from aggregate (not by group assignment) baseline and aggregate safety data for the study population.

### Research ethics approval

The Arizona State University Institutional Review Board has approved all protocols, data and safety monitoring plans, and study materials. All protocol amendments, other than minor administrative changes as defined by the NCCIH Guidance on Changes in Clinical Studies in Active Awards, will be submitted in a prospective manner to NCCIH except when necessary to protect the safety, rights, or welfare of subjects. Prior to submission to NCCIH, the proposed changes will be reviewed and approved by the IMC. IRB approval will not be sought until after NCCIH approval of the protocol amendment has been obtained.

### Informed consent

All consent documents will be obtained electronically, via Qualtrics, prior to the woman’s participation in the study (phase 1 or phase 2). The consent form will describe the purpose of the study, the procedures to be followed, and the risks and benefits of participation. A PDF copy will be emailed to the participants at their request.

### Timeline

See Table 5 for project timeline.

**Table 5:** Project timeline

Item	Year 1—months						Year 2—months						Year 3—months					
	1–2	3–4	5–6	7–8	9–10	11–12	13–14	15–16	17–18	19–20	21–22	23–24	25–26	27–28	29–30	31–32	33–34	35–36
Hire research personnel	X	X																
Continue to engage non-for profit partnerships		X	X	X	X	X												
Finalize manual of procedures for all protocols		X	X															
Finalize participant materials			X															
Finalize control group prescription				X														
Finalize Udaya yoga prescription				X														
Develop recruitment plans and materials				X	X													
Train research staff on study rocedures					X													
Modifications to Udaya.com (i.e., fidelity tracking, filming control group sessions)			X	X	X													
Obtain IRB approval						X												
Recruitment and enrollment							X	X	X	X								
Baseline data collection							X	X	X	X	X							
Post data collection								X	X	X	X	X						
Follow-up data collection									X	X	X	X	X					
Data analysis										X	X	X	X	X	X	X		
Manuscript preparation											X	X	X	X	X	X	X	X
Grant preparation										X	X	X	X	X	X	X	X	X

## Discussion

This RCT study will explore the feasibility and acceptability of a 12-week, home-based, online-streamed yoga intervention, with varying doses (low = 60 min/week; moderate = 150 min/week) among bereaved mothers who have experienced a stillbirth within 6 weeks to 24 months. To date, this is the first study to ascertain the preliminary effects of home-based, online-streamed yoga on reducing PTSD symptoms below the clinical threshold in mothers who have experienced the death of a baby to stillbirth. This study is particularly important not only because the risk for PTSD is nearly seven times the risk as compared to that for mothers with live births but also because there have only been two randomized control trials in the last 30 years aimed at improving emotional and mental health in these grieving mothers, which also affects surviving and subsequently born children. If feasible, the findings from this study will inform a full-scale trial to determine the effectiveness of home-based, online-streamed yoga to improve PTSD. Long-term, health care providers could use online yoga as a non-pharmaceutical, inexpensive resource to help women manage their symptoms after a stillbirth.

## Abbreviations

AE: Adverse event
ER: Emergency room
ERQ: Emotional Regulation Questionnaire
HIPAA: Health Insurance Portability and Accountability Act
IES-R: Impact of Event Scale-Revised
IMC: Independent Monitoring Committee
IRB: Institutional Review Board
LD: Low dose
MAAS: Mindful Attention Awareness Scale
MD: Moderate dose
NCCIH: National Center for Complementary and Integrative Health
NIH: National Institutes of Health
PGS: Perinatal Grief Scale
PHQ-9: Patient Health Questionnaire-9
PSQI: Pittsburgh Sleep Quality Index
PWMAQ: Past-week Modifiable Activity Questionnaire
RCT: Randomized controlled trial
SAE: Serious adverse event
SCS: Self-Compassion Scale
STAI: State-Trait Anxiety Inventory
STC: Stretch and tone control
